# Structural barriers in the context of opiate substitution treatment in Germany - a survey among physicians in primary care

**DOI:** 10.1186/1747-597X-8-26

**Published:** 2013-07-22

**Authors:** Bernd Schulte, Christiane Sybille Schmidt, Olaf Kuhnigk, Ingo Schäfer, Benedikt Fischer, Heiner Wedemeyer, Jens Reimer

**Affiliations:** 1 Centre for Interdisciplinary Addiction Research, Hamburg University, University Medical Centre Hamburg-Eppendorf, Martinistrasse 52, Hamburg, D-20246, Germany; 2 Centre for Applied Research in Mental Health and Addiction, Faculty of Health Sciences, Simon Fraser University, Vancouver, British Columbia, V6B 5K3, Canada; 3 Social & Epidemiological Research, Centre for Addiction and Mental Health, Toronto, Ontario, M5S 2S1, Canada; 4 Department of Gastroenterology, Hepatology and Endocrinology, Hannover Medical School, Carl-Neuberg-Strasse 1, Hannover, D-30625, Germany

**Keywords:** Barriers, Opiate substitution treatment, Primary care, Drug-related infectious diseases, Psychiatric care, Regulation, Health system, Germany

## Abstract

**Background:**

Opiate substitution treatment (OST) is the most widely used treatment for opioid dependence in Germany with substantial long-term benefits for the patient and for society. Due to lessened restrictive admission criteria, the number of registered OST patients in Germany has increased continuously in the recent years, whereas the number of physicians providing OST has remained constant. Previous data already indicated a deteriorating situation in the availability or quality of OST delivered and that structural barriers impede physicians in actively providing OST. The present survey among a sample of primary care physicians in Germany aimed to identify and assess potential structural barriers for the provision of health care in the context of OST.

**Methods:**

An anonymous written questionnaire was sent out to a sample of 2,332 physicians across Germany providing OST. Physicians contacted were identified through databases of the Federal State Chambers of Physicians and/or of the Federal Associations of Statutory Health Insurance Physicians. Data obtained were analysed descriptively.

**Results:**

The response rate was 25,5% and the majority of 596 physicians sampled viewed substantial problems in terms of the regulatory framework of OST care in the German context. Furthermore, financial remuneration, insufficient qualification, as well as inadequate interdisciplinary cooperation in the treatment of comorbidities of opiate substituted patients were regarded as problematic. The number of physicians providing OST in Germany is expected to substantially decrease in the near future.

**Conclusion:**

Despite less restrictive admission criteria for OST in Germany, the legal regulation framework for OST is still a limiting factor through raising concerns on the provider and consumer side to be unable to adhere to the strict rules. To avoid future shortages in the provision of OST care on the system level in Germany, revisions to the legal framework seem to be necessary. In regards to adequate care for drug use-related infectious diseases and psychiatric comorbidities commonly found in opiate substituted patients, efforts are required to improve professional qualifications of physicians providing OST as well as respective interdisciplinary collaboration.

## Introduction

The effectiveness of opiate substitution treatment (OST) and its substantial long-term benefits for patient and society in the treatment of opioid dependence are well documented. OST, e.g. with methadone or buprenorphine, improves the patients’ health and reduces illicit opiate use, decreases the risk of infections with blood-borne viruses, and reduces overdose-related deaths [1-6]. Recently, comparable findings were also shown for Germany [7,8]. Current prevalence estimations based on treatment, police and drug-related death data from 2011 suggest a population of up to 174,000 problem opiate users in Germany [9]. Heroin is the predominant opiate abused and nearby all problem opiate users are heroin users.

In Germany, OST is mainly delivered by office based GPs, in conjunction with community-based pharmacies; some OST delivery also occurs in specialized addiction clinics. In community-based treatment, the regulatory framework allows for take-home doses of up to seven days [10]. OST is considered the first-line treatment for severe chronic opioid dependence and its ultimate goal of OST in Germany is opioid abstinence [10]. To achieve this, the German OST concept includes protection of the patient’s health and social stabilization. The key inclusion criterion for OST is a main diagnosis of opiate dependence according to ICD-10. To qualify for the provision of OST in Germany, a physician has to meet specific qualification requirements for addiction therapy. OST physicians have to register each patient at the Federal Narcotics Control Board (Bundesopiumstelle) and are obliged to document all relevant patient and treatment data (e.g. diagnoses, psychosocial counselling, frequencies and results of drug screenings and supervisions of additional use of psychotropic substances) [10]. In the past, violations against the German regulations by OST physicians resulted in prosecutions and/or occupational consequences like fines, suspended sentences and revocations of the medical license.

**Figure 1:**
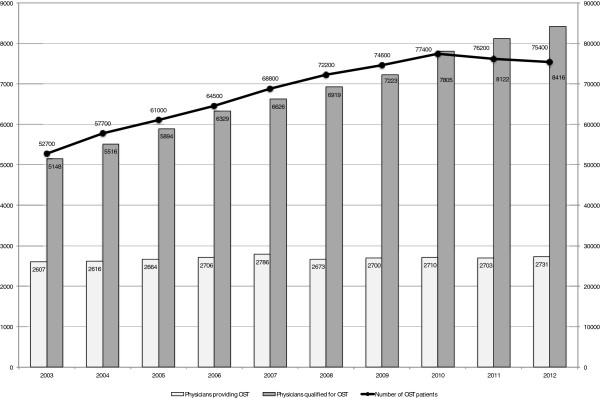
OST in Germany - Number of patients in OST, number of physicians qualified for OST and number of physician providing OST in the period 2003–2012.

OST is the most widely used treatment for opioid dependence in Germany. The number of registered OST patients increased by almost 44% - from 52.700 to 75.400 – between 2003 and 2012 (Figure 1) [11]. Similarly, the number of physicians certified for OST increased continuously from 5.148 in 2003 to 8.416 in 2012 [11]. The number of physicians actively providing OST care, however, has remained broadly constant (from N = 2,607 in 2003 to N = 2.731 in 2012) (Figure 1). In 2012, less than one third of the licensed physicians were providing OST. As a consequence, the average ratio of OST patients to care provider increased from 20 in 2003 to more than 27 in 2012.

Previous data indicate that current German regulations do not stimulate physicians to engage in OST [12-15]. In particular, the strict legal requirements of the German Narcotics Act (Betäubungsmittelgesetz, BtMG) and the respective regulations for the prescription of narcotics are regarded as a major structural barrier [12-15]. Especially the integration of the German Narcotics Act in the penal law constitutes a main reason for a legal uncertainty for providers [14]. In a pilot survey, we had already identified further structural barriers for the provision of OST in Germany, such as insufficient remuneration, inadequate availability of psychosocial support and lack of interdisciplinary cooperation in the treatment of drug-related infectious diseases and psychiatric comorbidities [14]. One fifth of the physicians were dissatisfied with the current OST conditions, while virtually everybody saw need for improvement [14]. These findings corroborated the outcomes of a national survey including representatives of the state chambers of physicians from all sixteen states in Germany, which represent the interests of physicians in terms of professional policy and legislative procedures. Six of them stated that the overall conditions for the provision of OST have deteriorated in recent years. As a consequence, five chambers regarded the regional provision of OST in some rural areas as already considerably inadequate or insufficient [12]. This reflected the findings of a more recent survey, which showed that access to OST was deemed inadequate by opiate users in and out of treatment, as well as by current and former OST prescribers, especially outside of major cities [15].

Similar to a number of other countries, the prevalence of psychiatric and somatic comorbidities, especially drug related infectious diseases, among opiate addicts is high in Germany [16]. Therefore, OST in Germany is regarded as an integral health care model including also medical care for drug related infectious diseases and psychiatric comorbidities. Hence, a deteriorating situation in the availability or quality of OST provision would undermine the objectives of standards as defined in the German OST regulations.

In order to better understand the current landscape of, and understand key factors for future OST provision, the principal aim of this survey therefore was to identify and assess potential structural barriers for the provision of OST and the medical care for drug-related infectious diseases and psychiatric comorbidities in context of OST in Germany.

## Methods

In March 2009, an anonymous questionnaire was sent out to a sample of 2,332 OST physicians registered in databases of the Federal State Chambers of Physicians and/or of the Federal Associations of Statutory Health Insurance Physicians. The questionnaire covered personal and care setting characteristics, such as medical specialisation, medical experience, size and location of the medical practice environment, professional long-term prospects (e.g. career planning, expected time until retirement) and treatment standards in regard to the target population (i.e., opioid dependent patients). Further question items focused on barriers encountered in the provision of health care for opiate addicts and on recommendations to improve the current OST situation. We used closed-ended question items to assess previously identified structural barriers. For instance, regarding to structural barriers for OST, we asked: “Please rate to which extent the following aspects hinder your daily work in OST: (A) Strong degree of regulation; (B) High interdisciplinary requirements; (C) Legal consequences by violation of regulations; (D) Disproportion between effort & remuneration; (E) Inadequate psychosocial support”. Such items had to be rated on a 5-point-Likert-scale from, “strongly disagree” to, “strongly agree”.

Open-ended question items were used to identify further possible structural barriers and to assess their impact on the provision of OST. Free text fields were used for physician suggestions to improve the current OST care situation (e.g. “What opportunities do you see to improve the current situation of OST care in your region?”). Ratings of the regional medical treatment situation for opiate addicts were assessed using a 6-point-Likert-scale following the German academic grading system (from “1 = very good” to “6 = totally deficient”). Subgroup analyses were conducted based on the following categories: size of treatment unit‘(small = up to 10 substitution clients per year; medium = 11 to 40 substitution clients per year; large = more than 40 treatment cases per year) and, “practice localisation” (urban area = more than 20,000 population size vs. rural area = 20,000 or less). Open item responses were coded and categorised manually based on thematic analysis; descriptive statistical analyses of quantitative data were performed using SPSS, version 18.0. Group comparisons were conducted using either Pearsons chi-squared tests (Fisher’s exact test if more than 20% of expected cell sizes were smaller than five), independent sample t-tests or one-way ANOVAs, depending on scale levels and number of groups to be compared. P-values smaller than .05 were considered statistically significant.

## Results

### Characteristics of the responding physicians and their treatment facilities

596 physicians (response rate: 25.5%) with a mean experience in OST of 12.3 years (± 5.67) completed and returned the questionnaire. In the response sample, a higher percentage of general practitioners provided OST as compared to psychiatrists (83.7% vs. 8.1%; Table 1). Compared to treatment facilities in rural areas, facilities in urban locations showed a higher grade of interdisciplinary specialisation: The urban subsample (n = 408) comprised 68% respondents from general practices, 13% from interdisciplinary group practices, 9% were specialised in internal medicine, 7% were psychiatrists, and 3% had other medical specialisations (e.g. gynecologists). The subsample from rural areas (n = 187) comprised 81% general practitioners, 10% from interdisciplinary group practices, 5% internists, 3% psychiatrists and 2% others; χ^2^ (4, n = 595) = 11.08, p = .026. Small treatment units seem to be more common in rural areas, whereas over 80% of the large substitution treatment centres were located in urban areas; χ^2^ (2, n = 595) = 41.769, p < .001 (Table 1).

**Table 1:** Health care setting and service characteristics of study physician participants, total sample and by treatment unit size

***Item***	***Total (N = 596)***	***Treatment unit size***^***S***^			***Group difference***
		***Small (N = 130)***	***Medium (N = 209)***	***Large (N = 257)***	
*Treatment unit characteristics*					
Single practice*	54.0	60.8	53.6	51.0	*p* = .003
Urban*	68.5	49.2	65.4	80.9	*p* < .001
Number of medical practitioners per practice**	1.8 (±1.4)	1.6 (±0.9)	1.7 (±1.8)	1.9 (±1.1)	*n.s.*
Practices with at least one psychologist or social worker*	10.1	3.1	2.9	19.5	*p* < .001
Total number of employees (including nurses, etc.)**	5.7 (±3.7)	4.7 (±2.5)	5.3 (±3.5)	6.7 (±4.2)	*p* < .001
Addiction society member*	23.3	4.6	16.7	38.1	*p* < .001
Medical specialisation* (multiple answers)					
General practitioner	83.7	86.9	83.7	82.1	*n.s.*
Internal medicine	19.6	20.8	18.7	19.8	*n.s.*
Psychiatry	8.1	4.6	3.3	13.6	*p* < .001
Additional qualifications*					
Infectiology	3.2	0.8	1.4	5.8	*p* = .006
Psychotherapy	4.4	4.6	2.9	5.4	*n.s.*
*Opiate substitution treatment (OST)*					
Number of patients in OST**	56.4 (±75.0)	5.0 (±3.3)	26.6 (±8.9)	106.6 (±91.7)	*p* < .001
Physicians’ years in OST**	12.4 (±5.7)	9.4 (±6.0)	13.1 (±5.3)	13.4 (±5.4)	*p* < .001
Assessment of regional OST care situation^G^	3.5 (±1.5)	3.8 (±1.5)	3.5 (±1.4)	3.4 (±1.5)	*p* = .037
Deficient regional OST care situation***	27.4	34.4	27.5	23.8	*n.s.*
*Infectious diseases (HCV/HIV)*					
Regular HIV/HCV Testing*	69.1	54.7	72.6	79.8	*p* < .001
Provide HIV treatment***	25.4	16.1	23.5	31.1	*p* = .006
Provide HCV treatment***	51.7	32.8	49.3	62.9	*p* < .001
Assessment of regional HCV/HIV care situation for OST patients^G^	2.9 (±1.4)	2.8 (±1.4)	2.8 (±1.3)	3.0 (±1.4)	*n.s.*
Deficient regional HCV/HIV care situation***	15.3	13.7	11.7	19.0	*n.s.*
*Psychiatric co-morbidities*					
Estimated percentage of OST patients with psychiatric comorbidity**	34.9 (±26.9)	37.3 (±33.4)	32.4 (±23.5)	35.9 (±26.2)	*n.s.*
Treatment of psychiatric co-morbidities always/often***	64.5	55.9	58.4	73.8	*p* < .001
Treatment of psychiatric co-morbidities always/often w/o psychiatrist*** (n = 544)	61.4	53.7	56.9	69.7	*p* = .004

^*S*^*According to number of OST clients/year: small = up to 10; medium = 11 to 40; large = more than 40.*

^*G*^*Mean values ± SD of ratings from 1 = very good to 6 = totally deficient.*

* Percentages; ** Mean ± SD; *** yes, percentages.

### Opiate substitution treatment

**Figure 2:**
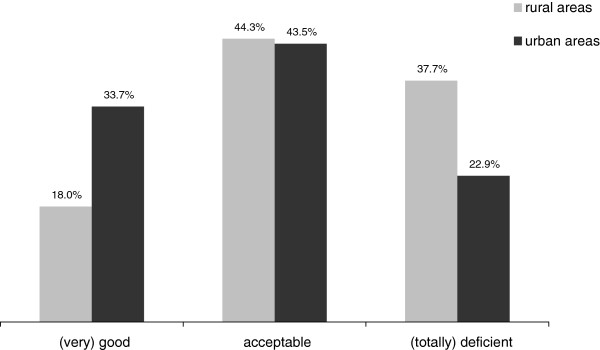
Assessment of regional OST care situation by OST physicians working in rural or urban areas.

The patient sample sizes to whom OST was provided in the treatment facilities represented by the responding physicians averaged 56.2 (±75.0) cases per year (Table 1); with 68.4 (±85.1) in urban facilities and 30.5 (±34.0) in rural treatment units; t (584.6) = −7.737, p < .001. The overall quality situation of OST provision was rated 3.5 (± 1.45) on a scale from 1 (very good) to 6 (totally deficient); overall quality was rated better by respondents based in urban locations: 3.3 ±1.4 urban vs. 3.9, ±1.5 rural; t (588) = 4.606, p < .001. One third of physicians working in rural areas rated the situation as “deficient” or “totally deficient”; less than 20% of this subgroup assessed the provision of OST in their region as “good” or “very good” (Figure 2).

More than 80% of respondents assessed the high level of OST regulations in conjunction with extensive administrative requirements as the main structural barriers, which “often” or “very often” complicated the daily work in OST (Table 2). Three out of four physicians regarded the financial remuneration as insufficient. Nearly 80% regarded the possible legal consequences in case of violation of OST regulations as inappropriate. Almost half of the physicians working in small OST facilities, as well as those in rural areas, perceived the regional availability of psychosocial support related to OST care to be inadequate. One third of the physician sample surveyed will retire – i.e. suspend their OST care activities - within the next 8 years.

**Table 2:** Structural barriers in the health care provision for substituted opiate addicts

***Items***	***All (N = 596)***	***Treatment unit size***^***S***^			***Group difference***
		***Small (N = 130)***	***Medium (N = 209)***	***Large (n = 257)***	
*Main barriers for OST (%; yes, often)*					
Strong degree of regulation	84.8	82.3	88.0	83.5	n.s.
High interdisciplinary requirements	29.7	24.6	32.7	29.8	n.s.
Legal consequences by violation of regulations	79.1	74.6	81.7	79,2	n.s.
Disproportion between effort & remuneration	75.0	76.9	80.3	69.8	*p* = .030
Inadequate psychosocial support	37.9	46.2	37.5	34.1	n.s.
*Main barriers for HIV treatment (%; yes, often)*					
Insufficient experience/qualification	59.7	58.5	64.1	56.8	n.s.
High treatment risks	26.3	26.2	29.2	24.1	n.s.
Budgetary reasons	29.9	35.4	31.6	25.7	n.s.
Lack of cooperation with regional experts	12.4	17.7	10.5	11.3	n.s.
Difficulties to integrate treatment in the daily routines	19.5	18.5	23.9	16.3	n.s.
*Main barriers for HCV treatment (%; yes, often)*					
Insufficient experience/qualification	34.2	39.2	39.7	27.2	*p* = .007
High treatment risks	19.5	20.8	21.5	17.1	n.s.
Budgetary reasons	23.7	28.5	28.2	17.5	*p* = .009
Lack of cooperation with regional experts	6.9	12.3	5.7	5.1	*p* = .021
Difficulties to integrate treatment in the daily routines	15.9	13.8	20.1	13.6	n.s.
*Main barrier for psychiatric care (%; yes, often)*					
Insufficient capacity to refer OST patients to psychiatrists	42.3	31.0	46.9	44.3	*p* = .012

^*S*^*According to number of OST clients/year: small = up to 10; medium = 11 to 40; large = more than 40.*

**Figure 3:**
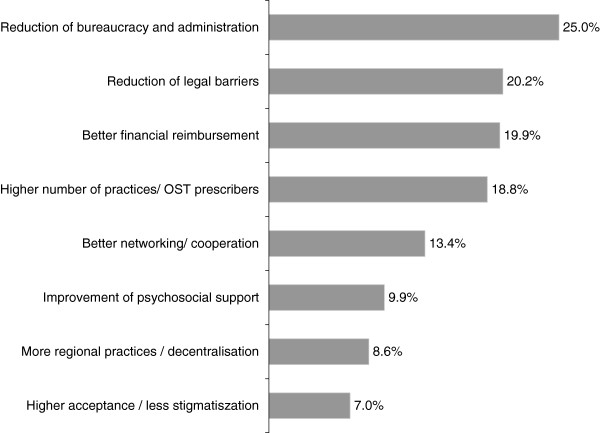
**Categories of suggestions from OST physicians to improve the current OST care situation in Germany.** Percentage of respondents, multiple suggestions were possible.

62.4% of our sample (n = 372) provided narrative suggestions for improvement of current conditions of OST care. The most predominant suggestions consisted of reducing bureaucracy and administrative responsibilities (25% of respondents), reducing legal barriers (20%) and improving the financial reimbursement (20%), which is in accordance with the identified structural barriers (Figure 3). Moreover, an overall higher number of OST prescribers considered improved cooperation and networking (13%) as important, and also the issues of the social acceptance and stigmatization of opioid addiction and/or OST were raised (7%). Other suggestions included the reduction of take-home regulations (3%) or increase/improvement of training programs (3%).

### Drug-related infectious diseases

One out of four treatment facilities surveyed offered antiretroviral treatment for human immunodeficiency virus (HIV) infections and half of the OST facilities offered antiviral hepatitis C (HCV) -therapy. On average, respondents assessed the overall situation for HIV/HCV care to be sufficient in their region; one in seven rated the situation as deficient. Insufficient professional experience and medical qualification were found to be main structural barriers in provision of HIV/HCV treatment care (Table 2). Further obstacles to providing HIV/HCV treatment care included limited financial compensation and the perceived risks and side effects of treatment. Accordingly, ways to increase the number of HCV/HIV treatments (selection from a list in the survey, multiple answers allowed) were seen as including a more adequate framework of financial reimbursement (HCV: 40.4%, HIV: 36.6%), designated HIV/HCV care education for physicians (HCV: 35.4%, HIV: 35.4%), and improved cooperation with HIV/HCV specialists (HCV: 32.7%, HIV: 30,7%). One in four physician respondents (26.0%) intended to withdraw from HCV/HIV treatment provision due to the above-mentioned barriers.

### Barriers in the provision of psychiatric care

A minority of OST physicians was trained as psychiatrists (Table 1), and one third of OST physicians (excluding psychiatrists) observed insufficient capacities in their regions for psychiatric referrals for OST patients. Consequentially, a high percentage of physicians without specialist training in psychiatry are treating psychiatric comorbidities in their OST patients. One in fourteen physicians considered cooperation with psychiatrist specialists to be adequate. Accordingly, the physician respondents suggested improving the local cooperation between psychiatrists and OST care providing physicians, increasing the number of psychiatrist specialists and implementing advanced training for psychiatrists in addiction medicine in the interest of an overall improvement of psychiatric care in OST. A further suggestion focused on an expanded availability of specialised psychiatric outpatient clinics as potential remedies for the observed deficits in psychiatric care. Only 1 out of 20 respondents did not see any need to improve the current psychiatric care situation for their patients.

## Discussion

This survey reviewed the practices and attitudes of physicians with longstanding experience in the provision of OST regarding the current status of health care for opiate addicts in their region, the impact of structural barriers on the provision of OST as well as medical care for drug-related infectious diseases and psychiatric comorbidities in Germany.

The majority of the sample agreed that different structural barriers currently hinder the provision of OST. In accordance with previous studies, the strict legal regulations of OST in combination with complex documentation requirements were identified as central obstacles [12,14,15]. Considering the empirical evidence of the effectiveness of OST [8] and the high number of OST patients enrolled in treatment in Germany [11] it can be assumed that the majority of treatment episodes occur without any major medical and legal problems. Hence, the extensive administrative requirements to meet the regulation requirements for the therapeutic use of OST medications – especially when compared to much more liberal approaches for other controlled medical substances or interventions - has to be questioned.

The highly complex German OST regulations are resulting in a legal uncertainty for OST providers, especially as several cases of violations have resulted in severely punitive consequences in their adjudications recently. No other medical field features legal regulations and requirements as strict - and potentially punitive in case of violations - as the field of OST. This might also be due to the historical development of OST regulations, which initially were based on the assumption that (opiate) addiction is a “criminal condition” and addicts are “morally inferior deviants” [17,18], resulting in compulsory, strict abstinence oriented treatment models.

Today, addiction is defined as a chronic, relapsing brain disease and addiction medicine and addiction research are more or less accepted professional medical specialties [19]. Based on this “medical paradigm” for opiate addiction treatment, the German regulations for OST have been revised several times in the last two decades. Especially, the revisions of minimum treatment admission requirements resulted in a substantive quantitative expansion of OST during this period. However, the limiting factor ‘OST regulations’ is still existing; physicians and patients are still concerned to be unable to adhere to the strict rules, which consequently impedes physicians to provide OST. This also raises the question of whether the primarily result-oriented (‘abstinence’) OST regulations deflect from the aim of health improvement, as they tangibly function as process-oriented obstacles.

The current OST regulations also seem to be a reason for the frequent responses suggesting that the financial remuneration for OST is inadequate. For instance, the regulations for opiate prescriptions in the context of OST usually require a weekly physician-patient consultation, while the Uniform Evaluation Scale (EBM), the compensation system of outpatient health care in Germany, only reimburses four consultations per quarter [12]. Furthermore, although OST should result in a substantial improvement and stabilization of the patient’s medical condition, the EBM only provides for a lump sum compensation per OST patient without additional compensation for the potential treatment of comorbidities.

Nevertheless, most OST prescribers in our study sample indicated that they provide comprehensive general medical care to their patients; for example, half of the prescribers offered pharmacotherapeutic HCV treatment, a rate that can be considered rather high in comparison with international standards. However, one third of the physicians surveyed felt insufficiently qualified to deliver more extensive HCV and/or HIV treatment. This might be reasonable, as the increased complexity of the current pharmacotherapeutic treatment regimens for these infectious diseases require specific medical expertise, which may exceed the medical qualifications and skills of GPs. In order to meet qualifications for provision of treatment for infectious diseases within the context of OST care, continuous specific medical education and intensified collaborations with regional specialists should be required [20]. Tailored approaches can optimise the HCV/HIV therapy for opiate addicts in primary care, e.g. integrated HCV/HIV care models [21-24]. A prerequisite for enhanced HCV/HIV care is regular screening and testing for infectious diseases among opiate dependent patients. In our sample, only 69% of OST prescribers offered regular HIV and Hepatitis C testing. Only 55% of those in the small treatment clinics offered this basic assessment, also suggesting that the adherence to the existing German clinical practice guidelines on the management of HCV infection is not optimal. In this guideline a regular HCV testing of every 12 months is required for OST patients without a previously diagnosed chronic HCV infection [25]. Targeted strategies to improve implementation of clinical practice guidelines might be helpful to increase related awareness among OST physicians [26,27].

The minority of OST physicians in Germany are specialised in psychiatry [14,15,28,29]. Given the high prevalence of psychiatric comorbidities among OST patients, a timely and continuous diagnostic and therapeutic psychiatric care plays a crucial role in this population yet cannot be covered solely by non-psychiatrist OST physicians [8]. The German health care system is not geared towards the high rates of - commonly complex - psychiatric comorbidities in OST patients, as also a successful treatment of substance-related disorders are required before psychiatric comorbidity care can be provided, often resulting in ‘revolving door’ dynamics between OST and psychiatric care. Furthermore, due to an increasing demand for psychiatric care in Germany, there is a general lack of psychiatric capacity in private practices and long waiting lists exist in some regions. Considering the high number of patients treated by non-psychiatrist physicians and the lack of referral options to psychiatrists, regional efforts for more psychiatric training of OST physicians and a closer cooperation with the psychiatric care sector should be established. This is also relevant in regard to the treatment of (psychiatric) side-effects in pharmacotherapeutic HIV and HCV therapies [30,31]. In turn, a closer cooperation between OST physicians and psychiatrists might therefore also result in an increased uptake of HCV/HIV treatments.

Another structural obstacle concerns the insufficient availability of OST-related psychosocial services, i.e. in rural regions. According to the new directives of the Federal Medical Council, the provision of psychosocial treatment and support remains a prerequisite for the provision of OST, but type and extent of care can vary according to patient needs [32]. This case-by-case decision might result in a more effective allocation of psychosocial care and might be helpful to shift resources especially to those OST patients, which require more psychosocial support. It would, however, be unrealistic to expect that a case-by-case allocation of OST-related psychosocial care will result in a substantial improvement of the overall provision in Germany. In general, there is a lack of long-term data on the effectiveness of different psychosocial interventions and their utilization in the context of OST in Germany [8].

Due to the relatively high average age of OST physicians [12], almost one-third of active OST physicians in the present sample are expected to retire within the next decade. Especially rural areas will then face a further aggravated situation of existing shortcomings and gaps in OST provision [12,15]. One option to compensate for the decrease in OST treatment resources could be to expand existing OST capacity into larger specialised substitution treatment centres (SSCs) and to allocate more patients to these units. Statistics of the German Federal Institute for Drugs and Medical Devices for the years 2006 to 2011 already indicate such a trend towards larger treatment centres. In 2011, half of all OST patients were treated in 15% of all OST treatment units [11].

However, a tendency towards the utilization of larger SSCs also has potential limitations. First, not all opiate addicts are attracted to specialised drug treatment centres and prefer OST care to be provided by a local GP [15]. Second, a tendency towards larger and concentrated treatment centres would also affect the OST availability in rural areas, as SSCs are mainly located in urban areas, and might therefore result in further geographic disparities in health care availability and clinical outcomes, and thus undermine the defined aims of OST in Germany. Furthermore, compared to SSCs, most GPs in Germany provide OST mainly to a limited number of patients within their general health care practice. These GPs may have limited time and space, or may not even want to provide OST to more patients. Interview data from former and current OST prescribers in Australia indicated that the willingness of GP’s to provide OST might be influenced by their perceived ability to control the number of OST patients [33]. In Germany, limited care resources and capacity required to integrate OST into their practice were found to be a main reason for the suspension of OST care among those who completely terminated the provision of OST services [15]. In the past, the relatively large number of GPs involved in OST care ensured a high diversification and OST coverage in Germany; such office-based approaches were found to be an effective facilitator for OST availability and utilization [34].

The present survey features some potential methodological limitations, including potential issues in regards to sampling. The study’s response rate of 25.5% was quite low, and thus may have introduced the possibility of selection bias into the responses received. In addition, the study relied on self-report data, none of which were independently verified; factors like social desirability may have influenced the content of responses, although the measures taken to protect the study participants’ identity (e.g. anonymity) can reasonably be expected to prevent such effects. Furthermore, the participants in the present survey were more likely those specialised in OST, as the proportion of large treatment centres represented was more than 2.5 times higher than in the representative sample of OST treatment units by Wittchen et al. [29]. Therefore, results are not generalizable beyond the specific study sample.

## Conclusion

Our survey demonstrated the existence of structural barriers in the provision of OST, and showed that these obstacles considerably limit the overall provision and uptake of OST. These structural barriers are relevant in regards to the key fact that the majority of opiate addicts in Germany still remain out of treatment, either by choice or due to limited treatment access [10,15]. The impact of the current structural conditions for OST care on physicians’ decisions to provide OST is notably strong: in the recent survey by Stöver et al., 82% of the physicians licensed for but not actively providing OST, reported that they had provided OST previously. The majority had terminated OST due to the difficult experiences with the existent regulatory framework [15].

Innovative approaches to overcome the identified structural barriers are needed and should involve meaningful adjustments to the legal and regulatory framework for OST in Germany. Recent revisions to the regulations for the prescription of narcotics might be helpful to ensure a continuous provision of OST in cases were OST physicians are not acutely available. However, to further improve current OST conditions, general revisions of also the philosophical scope of the present OST regulations are required, as the categorical abstinence-orientation of OST in Germany increases the risk of premature termination of OST as well as patient mortality [7,8]. While some described systemic barriers (i.e. lack of training, lack of support services, adequate financial compensation) from studies conducted outside of Germany are comparable to our findings [35-38], our study identified strict legal regulations as a principal structural barrier for OST in Germany.

## Competing interests

The study was funded in part by an unrestricted educational grant from Essex Pharma Germany. Essex Pharma had no role in the generation or approval of the manuscript.

All authors declare that they have no competing interest with respect to the content of this manuscript. BS reported receiving financial compensation for consultation and/or lectures from Lundbeck and Janssen-Cilag. HW reported receiving financial compensation for consultation and/or lectures from Roche, MSD, Bristol Myers Squibb, and Gilead. JR reported receiving financial compensation for consultation/lectures/unrestricted educational grants from Lundbeck, Molteni, MSD, Reckitt-Benckiser, Roche, and Sanofi-Aventis. All other authors reported no financial relationships.

## Authors’ contributions

BS and CSS performed the analyses of data collected, and composed initial drafts of the manuscript. OK, IS, BF, HW and JR contributed substantive content and crucial revisions to the manuscript. All authors have reviewed and approved the manuscript submitted.
